# Risk factors for plastic bronchitis in children with community-acquired pneumonia: a retrospective bronchoscopy-based study

**DOI:** 10.1186/s12890-026-04306-y

**Published:** 2026-04-24

**Authors:** Haiyan Qin, Xiangli Li, Qin Huang, Chang Peng

**Affiliations:** Department of Pediatrics, Guizhou Children’s Hospital, Affiliated Hospital of Zunyi Medical University, Zunyi, Guizhou China

**Keywords:** Pathogen, Clinical features, Prognosis, Bronchoscope, Children

## Abstract

**Objective:**

To investigate the pathogen distribution and clinical characteristics of pediatric community-acquired pneumonia (CAP) based on bronchoscopy findings, and to analyze the risk factors for plastic bronchitis (PB).

**Methods:**

A retrospective analysis was conducted on the clinical data of 761 children with community-acquired pneumonia (CAP) who underwent bronchoscopy in our hospital from January to December 2024. Based on the presence or absence of plastic casts observed under bronchoscopy, the children were divided into a plastic bronchitis (PB) group (*n* = 100) and a non-PB group (*n* = 661). Etiological results, clinical manifestations, laboratory findings, and prognostic indicators were collected for both groups. Univariate and multivariate logistic regression analyses were performed to identify independent risk factors for the development of plastic bronchitis.

**Results:**

Among the 761 children, the overall pathogen detection rate was 72.4% (551/761). Single pathogen infections were identified in 470 cases (61.8%), among which *Mycoplasma pneumoniae* (MP) was the most prevalent (356 cases, accounting for 75.7% of single infections), followed by *Haemophilus influenzae* (44 cases, 9.4%) and *Streptococcus pneumoniae* (37 cases, 7.9%). Mixed infections were observed in 85 cases (11.2%), with the most common combinations being *S. pneumoniae* + *H. influenzae* (17 cases), MP + *H. influenzae* (14 cases), and *S. pneumoniae* + MP (13 cases).

A total of 100 children (13.1%) were classified into the plastic bronchitis (PB) group and 661 (86.9%) into the non-PB group. Compared to the non-PB group, children in the PB group were significantly older [60 months vs. 36 months], had a higher proportion of left-sided pneumonia (23.0% vs. 11.2%), a lower proportion of bilateral pneumonia (55.0% vs. 67.0%), a lower rate of hospital stay ≤1 week (18.0% vs. 31.8%), and a higher rate of requiring ≥2 bronchoscopic procedures (30.0% vs. 8.9%); all differences were statistically significant (P < 0.05). Multivariate logistic regression analysis revealed that older age (OR = 1.009, 95% CI: 1.003–1.015, P = 0.002), left-sided pneumonia (OR = 2.114, 95% CI: 1.080–4.138, P = 0.029), and isolated Mycoplasma pneumoniae infection (OR = 1.781, 95% CI: 1.015–2.868, P = 0.018) were independent risk factors for the development of plastic bronchitis. Conversely, the non-PB group had a significantly higher rate of hospital stay ≤1 week compared to the PB group (OR = 0.390, 95% CI: 0.225–0.677, P = 0.001).

**Conclusion:**

Mycoplasma pneumoniae was the predominant pathogen among children with CAP in this cohort. Older age, left-sided pneumonia, and M. pneumoniae infection were identified as independent risk factors for PB. Early recognition of these factors may facilitate timely bronchoscopic intervention and improve clinical outcomes.

**Supplementary Information:**

The online version contains supplementary material available at 10.1186/s12890-026-04306-y.

## Introduction

Community-acquired pneumonia (CAP) is one of the most common infectious diseases in childhood and a leading cause of hospitalization and mortality among children under five years of age [1, 2]. Despite advances in antimicrobial therapy and supportive care, pediatric CAP continues to be associated with high morbidity and a substantial disease burden, particularly in cases of severe pneumonia, which may lead to serious complications or even death [1, 3]. The diversity of pathogens and the growing challenge of antimicrobial resistance complicate the diagnosis and treatment of pediatric CAP. Therefore, elucidating the pathogen profile and clinical characteristics of the disease is of great significance for guiding precision treatment [4, 5].

In recent years, with the widespread application of bronchoscopy in the field of pediatrics in China, this technique has played an increasingly important role in the diagnosis and treatment of community-acquired pneumonia (CAP) in children [6, 7]. Bronchoscopy not only allows for direct visualization of endobronchial lesions but also enables etiological detection through bronchoalveolar lavage fluid (BALF). Particularly in children with severe, refractory pneumonia, or those with poor radiographic resolution, this approach can improve pathogen detection rates and guide treatment [8, 9]. Furthermore, bronchoscopy can reveal a distinct pathological entity—plastic bronchitis (PB)—characterized by the formation of branching mucoid or plastic casts within the airways. These casts can cause airway obstruction, leading to respiratory distress and even respiratory failure, thus often necessitating bronchoscopic intervention [10, 11]. Multiple studies have confirmed that PB is not uncommon in pediatric CAP and is particularly associated with Mycoplasma pneumoniae infection [12–14].

While numerous studies have investigated the clinical characteristics of pediatric CAP [4, 15], large-sample studies based on bronchoscopy findings remain relatively limited. Moreover, there is a lack of systematic analysis regarding the incidence, etiological characteristics, and risk factors for PB. Additionally, the pathogenesis of PB is not yet fully understood, and early identification of its risk factors is crucial for guiding clinical intervention and improving patient outcomes.

Therefore, this study retrospectively analyzed the clinical data of 761 children with CAP who underwent bronchoscopy and bronchoalveolar lavage. The objectives were: (1) to analyze the distribution of pathogens in pediatric CAP; (2) to compare the clinical characteristics and outcomes between children with and without PB; and (3) to identify independent risk factors for the development of PB, thereby providing a clinical basis for early recognition and precision treatment of pediatric CAP.

## Materials and methods

### Patients

This retrospective study included pediatric patients diagnosed with community-acquired pneumonia (CAP) who underwent bronchoscopy at the Department of Pediatrics, Affiliated Hospital of Zunyi Medical University, between January 2024 and December 2024. The inclusion criteria were children diagnosed with CAP who received bronchoscopy and bronchoalveolar lavage during hospitalization. The exclusion criteria were as follows: (1) age > 14 years; (2) incomplete clinical data; and (3) patients who had undergone bronchoscopy at other medical institutions prior to admission”修改为“The exclusion criteria were as follows: (1) age > 14 years; (2) incomplete clinical data, defined as the absence of key variables necessary for the primary analysis, including demographic characteristics (age or sex), radiological findings, bronchoscopic confirmation of plastic bronchitis, or etiological test results; such cases were excluded to ensure the validity and completeness of the regression analysis; and (3) patients who had undergone bronchoscopy at other medical institutions prior to admission. This study was approved by the Ethics Committee of the Affiliated Hospital of Zunyi Medical University (Approval No: KLL-2025-614). As this was a retrospective study and no personally identifiable information was involved, the requirement for informed consent was waived. CAP was diagnosed based on clinical symptoms, physical examination, and radiological findings according to the World Health Organization (WHO) guidelines and established pediatric CAP diagnostic criteria [15].

### Data collection

Clinical data were collected from the hospital electronic medical record system. The variables included demographic characteristics (age and sex), radiological findings from chest computed tomography (CT), bronchoscopic findings (including endobronchitis and plastic bronchitis), length of hospital stay, time to symptom relief after bronchoscopy, and the number of bronchoscopic procedures performed. Disease severity was classified based on established pediatric CAP criteria. Severe pneumonia was defined according to clinical guidelines as the presence of any of the following: respiratory distress (e.g., tachypnea, chest indrawing), hypoxemia (oxygen saturation < 92%), requirement for oxygen therapy or mechanical ventilation, or signs of systemic involvement such as altered consciousness or hemodynamic instability [15].

### Etiological detection

Etiological identification was performed using routine microbiological and molecular diagnostic methods. Bacterial pathogens were detected primarily through sputum culture. Sputum specimens were collected under sterile conditions and processed in the hospital microbiology laboratory according to standard microbiological procedures. Bacterial isolates were identified using conventional culture techniques. Potential contaminant organisms, such as coagulase-negative staphylococci (e.g., *Staphylococcus epidermidis* and *Corynebacterium species)*, were interpreted with caution. These organisms were only considered potential pathogens when supported by clinical correlation, including compatible clinical presentation, radiological findings, and repeated or predominant growth in culture; otherwise, they were considered likely contaminants. Respiratory viral pathogens were detected using real-time polymerase chain reaction (PCR) assays based on nasopharyngeal swab specimens. The assays were performed using commercially available multiplex PCR kits (Sansure Biotech Inc., Changsha, China) according to the manufacturer’s instructions. The panel included common respiratory viruses such as respiratory syncytial virus (RSV), influenza A and B viruses, adenovirus, parainfluenza viruses, human rhinovirus. Detection of *Mycoplasma pneumoniae* infection was performed using serological testing. Peripheral venous blood samples were collected, and Mycoplasma pneumoniae-specific IgM antibodies were measured using a commercially available quantitative immunoassay kit (Zhuhai Livzon Diagnostics Inc., Zhuhai, China) according to the manufacturer’s instructions. The IgM antibody levels were expressed as titers, and a titer ≥ 1:40 was considered positive, based on the manufacturer’s recommended threshold. Polymerase chain reaction (PCR) testing for Mycoplasma pneumoniae was not routinely performed in our institution during the study period. Therefore, the diagnosis of MP infection in this study was primarily based on quantitative IgM serological results. It should be noted that serological detection of *Mycoplasma pneumoniae* (IgM) may have limited specificity in distinguishing acute from past infection.

Mixed infection was defined as the simultaneous detection of two or more pathogens in the same patient.

### Flexible bronchoscopy

Bronchoscopy was performed using a flexible fiberoptic bronchoscope by experienced pediatric bronchoscopists. The procedure was conducted under appropriate sedation and local anesthesia according to standard clinical protocols. Bronchoalveolar lavage (BAL) was performed in the affected bronchial segments, and lavage fluid was collected for microbiological analysis. The presence of plastic bronchitis was confirmed when branching bronchial casts were visualized and removed during bronchoscopy.

### Statistical analysis

Statistical analyses were performed using SPSS version 29.0 (IBM Corp., Armonk, NY, USA). Continuous variables were expressed as median and interquartile range [M (Q1, Q3)] and compared using the Mann–Whitney U test. Categorical variables were presented as numbers and percentages [n (%)] and compared using the chi-square test. Variables with *P* < 0.05 in the univariate analysis were included in the multivariate logistic regression model to identify independent risk factors for the development of plastic bronchitis. Odds ratios (OR) and 95% confidence intervals (CI) were calculated. All statistical tests were two-tailed, and *P* < 0.05 was considered statistically significant. It should be noted that some variables, such as length of hospital stay, are more likely to reflect disease severity or clinical outcomes rather than true baseline predictors of plastic bronchitis (PB). These variables were initially included in the regression model for exploratory purposes, given their clinical relevance. However, to address potential reverse causality, we performed additional sensitivity analyses excluding such variables to ensure the robustness of the identified risk factors.

## Results

### Etiological characteristics

A total of 761 children with community-acquired pneumonia (CAP) who underwent bronchoscopy and bronchoalveolar lavage were enrolled in this study. The overall pathogen detection rate was 72.4% (551/761). Among all cases, single pathogen infection was identified in 470 children (61.8%), co-infection with two pathogens in 85 children (11.2%), and co-infection with three pathogens in 10 children (1.3%), while pathogen detection was negative in 206 children (27.1%). Among single pathogen infections, *Mycoplasma pneumoniae* was the most prevalent, detected in 356 cases, accounting for 75.7% of single infections and 46.8% of the total study population. This was followed by *Haemophilus influenzae* (44 cases, 9.4% of single infections), *Streptococcus pneumoniae* (37 cases, 7.9%), *methicillin-resistant Staphylococcus aureus* (6 cases, 1.3%), and *Staphylococcus aureus* (5 cases, 1.1%). Viral pathogens were detected less frequently, with *respiratory syncytial virus* (RSV) being the most common (4 cases, 0.9%). Regarding co-infections, the combination of *Streptococcus pneumoniae* and *Haemophilus influenzae* was the most frequently observed (17 cases, accounting for 20.0% of all co-infections), followed by *Haemophilus influenzae* plus *Mycoplasma pneumoniae* (14 cases, 16.5%) and *Streptococcus pneumoniae* plus *Mycoplasma pneumoniae* (13 cases, 15.3%)(Table 1), it should be noted that some organisms, such as Staphylococcus epidermidis and Corynebacterium species, may represent colonization or contamination rather than true pathogens and should be interpreted with caution.

**Table 1:** Comparison of etiological distribution between children with and without plastic bronchitis [n(%)]

Pathogen type	Plastic bronchitis group (n=100)	Non-plastic bronchitis group (n=661)
Single pathogen infection	74 (74.0)	396 (59.9)
Mycoplasma pneumoniae	64 (64.0)	292 (44.2)
Haemophilus influenzae	5 (5.0)	39 (5.9)
Streptococcus pneumoniae	4 (4.0)	33 (5.0)
Escherichia coli	1 (1.0)	2 (0.3)
Methicillin-resistant Staphylococcus aureus	0	6 (0.9)
Staphylococcus aureus	0	5 (0.8)
Moraxella catarrhalis	0	5 (0.8)
Respiratory syncytial virus	0	4 (0.6)
Acinetobacter baumannii	0	3 (0.5)
Klebsiella pneumoniae	0	3 (0.5)
Enterobacter aerogenes	0	1 (0.2)
Pseudomonas aeruginosa	0	1 (0.2)
Corynebacterium amycolatum	0	1 (0.2)
Staphylococcus epidermidis	0	1 (0.2)
Co-infection (two pathogens)	6 (6.0)	69 (10.4)
Streptococcus pneumoniae + Haemophilus influenzae	2 (2.0)	15 (2.3)
Haemophilus influenzae + Mycoplasma pneumoniae	2 (2.0)	12 (1.8)
Streptococcus pneumoniae + Mycoplasma pneumoniae	0	12 (1.8)
Methicillin-resistant Staphylococcus aureus + Mycoplasma pneumoniae	1 (1.0)	3 (0.5)
Escherichia coli + Mycoplasma pneumoniae	1 (1.0)	0
Streptococcus pneumoniae + Respiratory syncytial virus	0	3 (0.5)
Pseudomonas aeruginosa + Mycoplasma pneumoniae	0	2 (0.3)
Methicillin-resistant Staphylococcus aureus + Klebsiella pneumoniae	0	2 (0.3)
Haemophilus influenzae + Methicillin-resistant Staphylococcus aureus	0	2 (0.3)
Klebsiella pneumoniae + Mycoplasma pneumoniae	0	1 (0.2)
Mycoplasma pneumoniae + Respiratory syncytial virus	0	1 (0.2)
Mycoplasma pneumoniae + Human rhinovirus	0	1 (0.2)
Streptococcus pneumoniae + Methicillin-resistant Staphylococcus aureus	0	1 (0.2)
Streptococcus pneumoniae + Pseudomonas aeruginosa	0	1 (0.2)
Streptococcus pneumoniae + Moraxella catarrhalis	0	1 (0.2)
Streptococcus pneumoniae + Escherichia coli	0	1 (0.2)
Haemophilus influenzae + Enterobacter cloacae	0	1 (0.2)
Haemophilus influenzae + Moraxella catarrhalis	0	1 (0.2)
Pseudomonas aeruginosa + Stenotrophomonas maltophilia	0	1 (0.2)
Methicillin-resistant Staphylococcus aureus + Acinetobacter baumannii	0	1 (0.2)
Haemophilus influenzae + Escherichia coli	0	1 (0.2)
Klebsiella pneumoniae + Escherichia coli	0	1 (0.2)
Staphylococcus aureus + Haemophilus influenzae	0	1 (0.2)
Pseudomonas aeruginosa + Enterobacter cloacae	0	1 (0.2)
Acinetobacter baumannii + Stenotrophomonas maltophilia	0	1 (0.2)
Moraxella catarrhalis + Respiratory syncytial virus	0	1 (0.2)
Haemophilus influenzae + Respiratory syncytial virus	0	1 (0.2)
Co-infection (three pathogens)	1 (1.0)	9 (1.4)
Haemophilus influenzae + Streptococcus pneumoniae + Adenovirus	0	2 (0.3)
Mycoplasma pneumoniae + Streptococcus pneumoniae + Haemophilus influenzae	0	1 (0.2)
Streptococcus pneumoniae + Methicillin-resistant Staphylococcus aureus + Haemophilus influenzae	0	1 (0.2)
Streptococcus pneumoniae + Escherichia coli + Influenza B virus	0	1 (0.2)
Streptococcus pneumoniae + Haemophilus influenzae + Respiratory syncytial virus	0	1 (0.2)
Streptococcus pneumoniae + Acinetobacter baumannii + Haemophilus influenzae	0	1 (0.2)
Streptococcus pneumoniae + Klebsiella pneumoniae + Haemophilus influenzae	0	1 (0.2)
Haemophilus influenzae + Methicillin-resistant Staphylococcus aureus + Pseudomonas aeruginosa	0	1 (0.2)
Streptococcus pneumoniae + Pseudomonas aeruginosa + Haemophilus influenzae	1 (1.0)	0
Negative	19 (19.0)	187 (28.3)

### Comparison of clinical characteristics between the two groups

Univariate analysis revealed significant differences in several clinical parameters between the plastic bronchitis (PB) group and the non-PB group (Table 2). No significant difference was observed in gender distribution between the two groups (male proportion: 56.0% vs. 63.1%, *P* = 0.173). The median age of children in the PB group was significantly higher than that in the non-PB group [60 (36, 84) months vs. 36 (12, 60) months, *P* < 0.001]. Regarding radiographic findings, the proportion of left-sided pneumonia was significantly higher in the PB group compared to the non-PB group (23.0% vs. 11.2%, *P* = 0.001), whereas the proportion of bilateral pneumonia was significantly lower (55.0% vs. 67.0%, *P* = 0.018). No significant difference was found in the proportion of right-sided pneumonia between the two groups (22.0% vs. 21.8%, *P* = 0.961). However, In terms of disease severity, the PB group exhibited a slightly higher proportion of severe pneumonia compared to the non-PB group; however, this difference did not reach statistical significance (43.0% vs. 37.1%, *P* = 0.254). Therefore, disease severity was not included in the multivariate logistic regression model (Table 2). The PB group demonstrated significantly poorer outcomes across multiple prognostic indicators compared to the non-PB group (Table 2). Specifically, the proportion of children with a hospital stay ≤ 1 week was significantly lower in the PB group than in the non-PB group (18.0% vs. 31.8%, *P* = 0.005), indicating that children with plastic bronchitis experienced prolonged hospitalization. However, no significant difference was observed between the two groups regarding the proportion with a hospital stay ≤ 2 weeks (86.0% vs. 87.6%, *P* = 0.654). Furthermore, there were no significant differences in symptom relief rates at 2 days and 3 days post-bronchoscopy between the two groups (*P* > 0.05). Notably, the proportion of children requiring ≥ 2 bronchoscopic interventions was significantly higher in the PB group compared to the non-PB group (30.0% vs. 8.9%, *P* < 0.001) (Table 2). Patients in the PB group had a longer hospital stay and required more bronchoscopic interventions compared to the non-PB group. However, these findings should be interpreted with caution, as they are likely inherent to the clinical course and management of PB rather than independent predictors, and may therefore reflect consequences rather than causes of the condition.

**Table 2:** Comparison of clinical characteristics and prognostic indicators between children with and without plastic bronchitis

Characteristic	Plastic bronchitis group (n=100)	Non-plastic bronchitis group (n=661)	χ²/Z	P-value
Male gender	56 (56.0)	417 (63.1)	1.854	0.173
Age (months)	60 (36-84)	36 (12-60)	-5.737	<0.001
Bilateral pneumonia	55 (55.0)	443 (67.0)	5.549	0.018
Right-sided pneumonia	22 (22.0)	144 (21.8)	0.002	0.961
Left-sided pneumonia	23 (23.0)	74 (11.2)	10.883	0.001
Severe pneumonia	43 (43.0)	245 (37.1)	1.301	0.254
Hospital stay ≤1 week	18 (18.0)	210 (31.8)	7.849	0.005
Hospital stay ≤2 weeks	86 (86.0)	579 (87.6)	0.2	0.654
Symptom relief within 2 days post-bronchoscopy	86 (86.0)	584 (88.4)	0.456	0.499
Symptom relief within 3 days post-bronchoscopy	96 (96.0)	637 (96.4)	0.033	0.855
Bronchoscopy performed ≥2 times	30 (30.0)	59 (8.9)	37.353	<0.001

### Multivariate logistic regression analysis of risk factors for plastic bronchitis

Variables with *P* < 0.05 in the univariate analysis were included in the multivariate logistic regression model. Variables that were clearly consequences of PB (e.g., number of bronchoscopic procedures) were excluded. Some variables that may reflect disease progression (e.g., length of hospital stay) were retained in the initial model for exploratory analysis but were further evaluated in sensitivity analyses to minimize potential bias due to reverse causality. The results indicated that: revealed that older age (OR = 1.009, 95% CI: 1.003–1.015, *P* = 0.002), left-sided pneumonia (OR = 2.114, 95% CI: 1.080–4.138, *P* = 0.029), and isolated *Mycoplasma pneumoniae* infection (OR = 1.781, 95% CI: 1.015–2.868, *P* = 0.018) were independent risk factors for the development of plastic bronchitis. Specifically, each one-month increase in age was associated with a 0.9% increase in the risk of PB; children with left-sided pneumonia had a 2.1-fold higher risk of PB compared to those with pneumonia in other locations; and children with isolated *M. pneumoniae* infection had a 1.8-fold higher risk of PB compared to those without isolated *M. pneumoniae* infection. Conversely, A hospital stay ≤ 1 week was associated with a lower likelihood of PB (OR = 0.390, 95% CI: 0.225–0.677, *P* = 0.001). However, given that hospital stay is more likely to reflect disease progression and severity rather than serve as a true predictor, this finding should be interpreted with caution (Table 3). We further included all cases with *M. pneumoniae* infection in the regression analysis, and the results were consistent with those obtained from the analysis that included only isolated *M. pneumoniae* infection (Supplementary Table 1). In addition, due to the limited number of plastic bronchitis cases caused by other pathogens, the results might have been unstable; therefore, no specific analysis was performed for these pathogens.

**Table 3:** Univariate and multivariate logistic regression analysis of risk factors for plastic bronchitis

Characteristic	Univariate analysis		Multivariate analysis	
	OR(95%CI)	P-value	OR(95%CI)	P-value
Age	1.012(1.007-1.017)	< 0.001	1.009(1.003-1.015)	0.002
Bilateral pneumonia	0.601(0.393-0.921)	0.019	-	-
Left-sided pneumonia	2.369(1.402-4.004)	0.001	2.114(1.080-4.138)	0.029
Hospital stay ≤1 week	0.471(0.276-0.806)	0.006	0.390(0.225-0.677)	0.001
Mycoplasma pneumoniae infection	2.247(1.452-3.475)	< 0.001	1.781(1.015-2.868)	0.018

### Sensitivity analysis

Because hospital stay may be a consequence rather than a cause of PB, a sensitivity analysis excluding this variable was performed, this approach allowed us to distinguish true baseline predictors from variables reflecting disease course, thereby strengthening the validity of our conclusions. The associations between PB and the remaining risk factors (older age, left-sided pneumonia, and isolated *M. pneumoniae* infection) remained statistically significant, indicating that our primary findings were robust and not driven by collinearity with hospital stay(Supplementary Table 2).

## Discussion

Community-acquired pneumonia (CAP) remains one of the leading causes of morbidity in children worldwide [1, 4, 16]. Plastic bronchitis (PB), characterized by the formation of bronchial casts that obstruct the airway, is a relatively rare but potentially severe complication of pediatric pneumonia [17–19]. Early identification of children at high risk for PB is therefore crucial for timely clinical intervention. In the present study, we retrospectively analyzed 761 children with CAP who underwent bronchoscopy and explored the clinical characteristics associated with PB. Our findings demonstrated that older age, left-sided pneumonia, and Mycoplasma pneumoniae (MP) infection were independently associated with an increased risk of PB.

MP infection was identified as a major risk factor for PB in our study. MP is one of the most common pathogens causing CAP in school-aged children and has been increasingly associated with severe pulmonary complications [20–22]. Previous studies have reported that PB frequently occurs in children with MP pneumonia, particularly in severe or refractory cases [13, 23]. The underlying mechanism may involve excessive airway inflammation induced by MP infection. MP can cause epithelial cell injury, mucus hypersecretion, and fibrin exudation in the bronchial tree, leading to the formation of bronchial casts and airway obstruction [24, 25]. In addition, immune-mediated mechanisms such as cytokine overproduction and inflammatory cell infiltration may further promote the development of PB.

Our study also showed that older age was associated with an increased risk of PB. This finding is consistent with previous reports indicating that PB occurs more frequently in school-aged children with MP pneumonia [12, 26, 27]. Older children tend to develop stronger immune responses to MP infection, which may lead to more pronounced airway inflammation and mucus secretion. These exaggerated inflammatory responses may facilitate the formation of bronchial casts and contribute to airway obstruction. Similar observations have been reported in previous studies of MP-associated PB in children .

Interestingly, left-sided pneumonia was identified as another independent risk factor for PB in this study. Although the precise mechanism remains unclear, several factors may contribute to this association. Differences in bronchial anatomy, airway drainage patterns, and regional ventilation may influence the accumulation of inflammatory secretions within specific bronchial segments. In addition, unilateral pulmonary consolidation and localized airway inflammation have been reported to be associated with PB formation in children with severe pneumonia [12, 28, 29]. These findings suggest that localized inflammatory processes in the affected lung segments may promote mucus plugging and bronchial cast formation.

Notably, children in the PB group had a significantly lower rate of hospital stay ≤ 1 week compared to those in the non-PB group, a finding consistent with the regression analysis (OR = 0.390, *P* = 0.001). This may reflect both the greater severity of illness in the PB group, necessitating prolonged hospitalization, and the potential benefit of early identification of PB risk, which could facilitate timely intervention and shorten hospital stay. Furthermore, we observed that the proportion of children requiring ≥ 2 bronchoscopic procedures was significantly higher in the PB group, indicating that these patients often require repeated lavage for cast removal. Clinicians should therefore remain vigilant in such cases.

From a clinical perspective, our findings may contribute to improved risk stratification in children with CAP. Patients presenting with older age, left-sided pneumonia, and Mycoplasma pneumoniae infection may be at increased risk for developing plastic bronchitis and may benefit from closer monitoring and earlier consideration of bronchoscopic evaluation. While these findings are not sufficient to directly inform clinical guidelines, they highlight potential high-risk features that could be incorporated into future prospective studies or clinical decision-making frameworks aimed at optimizing the timing of bronchoscopy and improving patient outcomes.

## Conclusion

In conclusion, this study found that the incidence of plastic bronchitis in children with community-acquired pneumonia was 13.1%, with *Mycoplasma pneumoniae* being the most significantly associated pathogen. Age, left-sided pneumonia, and *MP* infection were identified as risk factors for the development of PB, which was also associated with prolonged hospitalization. Early recognition of these risk factors may facilitate timely bronchoscopic intervention and improve clinical management in children with CAP. Future prospective studies are warranted to validate these findings and further explore the underlying molecular mechanisms.

### Limitations

Several limitations of this study should be acknowledged. First, an important methodological consideration in this study is the distinction between predictors and outcome-related variables. For example, length of hospital stay is more likely to be influenced by disease severity and the occurrence of PB rather than acting as a true predictor. Although it was included in the initial regression model due to its clinical relevance, we performed sensitivity analyses excluding this variable. The main findings remained unchanged, supporting the robustness of the identified risk factors and reducing concerns about reverse causality. Second, the detection of Mycoplasma pneumoniae infection relied primarily on quantitative serological testing (IgM), without routine confirmation by polymerase chain reaction (PCR), which is considered the preferred diagnostic method for acute MP infection. Although the use of quantitative IgM titers may improve specificity compared with qualitative assays, IgM antibodies may persist after infection and may be subject to cross-reactivity. In addition, the absence of paired serological testing (e.g., a fourfold rise in IgG titers) further limits diagnostic accuracy. Therefore, the prevalence of MP infection in this cohort may have been overestimated, which could have influenced the observed association between MP infection and plastic bronchitis. These findings should be interpreted with caution and validated in future studies using PCR-based methods. In addition, some bacterial pathogens identified through sputum culture, particularly organisms such as Staphylococcus epidermidis and Corynebacterium species, may represent colonization or contamination rather than true causative agents of pneumonia. Although efforts were made to interpret microbiological results in conjunction with clinical findings, misclassification cannot be completely excluded, which may have influenced the reported pathogen distribution.

## Supplementary Information

Supplementary Material 1.

Supplementary Material 2.

Supplementary Material 3.

Supplementary Material 4.

Supplementary Material 5.

## Data Availability

The datasets used and/or analyzed during the current study available from the corresponding author on reasonable request.

## Abbreviations

BAL: Bronchoalveolar lavage
BALF: Bronchoalveolar lavage fluid
CAP: Community-acquired pneumonia
CI: Confidence interval
CT: Computed tomography
IgM: Immunoglobulin M
MP: Mycoplasma pneumoniae
MRSA: Methicillin-resistant Staphylococcus aureus
OR: Odds ratio
PB: Plastic bronchitis
PCR: Polymerase chain reaction
RSV: Respiratory syncytial virus
WHO: World Health Organization
